# Supplementary material for: ACRBP (Sp32) is involved in priming sperm for the acrosome reaction and the binding of sperm to the zona pellucida in a porcine model
Source: PLoS One. 2021 Jun 4;16(6):e0251973. doi: 10.1371/journal.pone.0251973 (PMC8177411; doi:10.1371/journal.pone.0251973)
Supplement: S1 Table — (PDF) [file pone.0251973.s001.pdf]

Supporting information files

S1 Table. The effect of anti-phosphotyrosine and anti-ACRBP antibodies upon sperm-ZP binding

Experimental Groups	Treatments	No. of zona-bound sperm		
		First time	Second time	Third time
Anti-phosphotyrosine antibodies	No antibody	69.3	52.4	52.4
	Blocking peptide IgG	58.6	50.1	49.8
	Anti-phosphotyrosine antibodies	33.6	32.7	27.1
Anti-ACRBP antibodies	No antibody	96.9	69.4	69.3
	Pre-immune rabbit IgG	92.3	62.9	58.6
	Anti-ACRBP antibodies	45.6	46.1	33.6

å
