# Supplementary material for: ACRBP (Sp32) is involved in priming sperm for the acrosome reaction and the binding of sperm to the zona pellucida in a porcine model
Source: PLoS One. 2021 Jun 4;16(6):e0251973. doi: 10.1371/journal.pone.0251973 (PMC8177411; doi:10.1371/journal.pone.0251973)
Supplement: S2 Table — (PDF) [file pone.0251973.s002.pdf]

**S2 Table. The effect of anti-phosphotyrosine# and anti-ACRBP antibodies upon fertilization**

| Experimental Groups | Treatments | No. of inseminated oocytes | Total Fertilization rate (%) | | | | |
|---|---|---|---|---|---|---|---|
| | | | First time | Second time | Third time | Fourth time | Fifth time |
| Anti-phosphotyrosine antibodies | No antibody | 64 | 58.3 | 68.4 | 77.8 | 80 | NA |
| | Blocking peptide IgG | 60 | 77.8 | 47.4 | 62.5 | 71.4 | NA |
| | Anti-phosphotyrosine antibodies | 55 | 18.8 | 28.6 | 46.7 | 40 | NA |
| Anti-ACRBP antibodies | No antibody | 67 | 100 | 60 | 68.4 | 58.3 | 80 |
| | Pre-immune rabbit IgG | 63 | 45.5 | 73.3 | 50 | 61.5 | 75 |
| | Anti-ACRBP antibodies | 73 | 33.3 | 23.1 | 44 | 40 | 33.3 |
