# Supplementary material for: ACRBP (Sp32) is involved in priming sperm for the acrosome reaction and the binding of sperm to the zona pellucida in a porcine model
Source: PLoS One. 2021 Jun 4;16(6):e0251973. doi: 10.1371/journal.pone.0251973 (PMC8177411; doi:10.1371/journal.pone.0251973)
Supplement: S3 Table — (PDF) [file pone.0251973.s003.pdf]

S3 Table. The influence of anti-phosphotyrosine# and anti-ACRBP antibodies upon sperm capacitation and the acrosome reaction

Experimental Groups	Treatments	Capacitation ratio (%)					
		First time	Second time	Third time	Fourth time	Fifth time	Sixth time
Anti-phosphotyrosine antibodies	No antibody	43.4	39	36	23.3	NA	NA
	Blocking peptide IgG	50.9	45	36	10	NA	NA
	Anti-phosphotyrosine antibodies	30	20.6	22.8	8	NA	NA
Anti-ACRBP antibodies	No antibody	54	43.4	39	43.1	37.5	NA
	Pre-immune rabbit IgG	39	60.4	46.8	33.2	40.7	NA
	Anti-ACRBP antibodies	25.5	35.6	21.7	12.2	24.1	NA
Experimental Groups	Treatments	Acrosome reaction ratio (%)					
		First time	Second time	Third time	Fourth time	Fifth time	Sixth time
Anti-phosphotyrosine antibodies	No antibody	50	45.1	41.3	NA	NA	NA
	Blocking peptide IgG	30.8	27	30.7	NA	NA	NA
	Anti-phosphotyrosine antibodies	35	27.3	25.7	NA	NA	NA
Anti-ACRBP antibodies	No antibody	30.5	45.1	41.5	42.2	48	44.8
	Pre-immune rabbit IgG	28.5	33.7	36.5	51.3	40.8	38.3
	Anti-ACRBP antibodies	16	35	24.7	33.5	36.5	10.5
