# Supplementary material for: ACRBP (Sp32) is involved in priming sperm for the acrosome reaction and the binding of sperm to the zona pellucida in a porcine model
Source: PLoS One. 2021 Jun 4;16(6):e0251973. doi: 10.1371/journal.pone.0251973 (PMC8177411; doi:10.1371/journal.pone.0251973)
Supplement: S4 Table — (PDF) [file pone.0251973.s004.pdf]

S4 Table. The impact of anti-ACRBP on the acrosome reaction induced by thapsigargin and A23187

**First time**	Acrosome reaction ratio (%)				
Culture period	Treatments	Medium only	Dimethyl Sulfoxide	A23187	Thapsigargin
1 hour	No antibody	48.23	42.3	85.2	70
	Pre-immune rabbit IgG	56	41.3	90.6	64.1
	Anti-ACRBP antibodies	32.3	46.9	77	48.3
4 hours	No antibody	64.7	56.4	93.2	66.8
	Pre-immune rabbit IgG	51	71.8	78.4	79
	Anti-ACRBP antibodies	18.4	26.5	69.4	34.3

**Second time**	Acrosome reaction ratio (%)				
Culture period	Treatments	Medium only	Dimethyl Sulfoxide	A23187	Thapsigargin
1 hour	No antibody	41.5	45.4	81.4	82.4
	Pre-immune rabbit IgG	42.9	40.4	88.5	82.5
	Anti-ACRBP antibodies	41	43.8	82.7	52
4 hours	No antibody	59	46.9	83.5	82.5
	Pre-immune rabbit IgG	58.7	54.1	79.8	74.6
	Anti-ACRBP antibodies	41	43.8	82.7	52

**Third time**	Acrosome reaction ratio (%)				
Culture period	Treatments	Medium only	Dimethyl Sulfoxide	A23187	Thapsigargin
1 hour	No antibody	23.7	30.7	65.9	67.9
	Pre-immune rabbit IgG	28.3	31.8	76.8	74.3
	Anti-ACRBP antibodies	22.8	28.1	73.3	40.5
4 hours	No antibody	39.4	32.4	78.5	68
	Pre-immune rabbit IgG	39.4	23.5	68.6	73
	Anti-ACRBP antibodies	18.4	26.5	69.4	34.3
