# Supplementary material for: ACRBP (Sp32) is involved in priming sperm for the acrosome reaction and the binding of sperm to the zona pellucida in a porcine model
Source: PLoS One. 2021 Jun 4;16(6):e0251973. doi: 10.1371/journal.pone.0251973 (PMC8177411; doi:10.1371/journal.pone.0251973)
Supplement: S5 Table — (PDF) [file pone.0251973.s005.pdf]

S5 Table. The effect of anti-ACRBP on the AR induction of boar sperm by soluble zona pellucida

**First time**	Acrosome reaction ratio (%)				
Treatments	Medium only	Dimethyl Sulfoxide	A23187	Thapsigargin	Soluble zona pellucida
No antibody	17.9	13	52.5	56.5	42.5
Pre-immune rabbit IgG	5.8	10.4	38.9	50.4	34.8
Anti-ACRBP antibodies	11	9.9	45	30.2	13

**Second time**	Acrosome reaction ratio (%)				
Treatments	Medium only	Dimethyl Sulfoxide	A23187	Thapsigargin	Soluble zona pellucida
No antibody	18.2	16.8	40.4	50	33
Pre-immune rabbit IgG	20.3	15.8	42	49.2	33
Anti-ACRBP antibodies	5.7	10.1	36.4	21.5	22.4

**Third time**	Acrosome reaction ratio (%)				
Treatments	Medium only	Dimethyl Sulfoxide	A23187	Thapsigargin	Soluble zona pellucida
No antibody	16.5	18.8	54.1	56.3	42.7
Pre-immune rabbit IgG	16	16.7	49.1	52.5	51.2
Anti-ACRBP antibodies	5.4	18.6	51.8	35	28.6

**Fourth time**	Acrosome reaction ratio (%)				
Treatments	Medium only	Dimethyl Sulfoxide	A23187	Thapsigargin	Soluble zona pellucida
No antibody	9.4	18	37.1	57.7	37.6
Pre-immune rabbit IgG	17.4	14.3	42.3	49.3	35.6
Anti-ACRBP antibodies	3.8	17.1	34.3	44.2	11.7
