# Supplementary material for: ACRBP (Sp32) is involved in priming sperm for the acrosome reaction and the binding of sperm to the zona pellucida in a porcine model
Source: PLoS One. 2021 Jun 4;16(6):e0251973. doi: 10.1371/journal.pone.0251973 (PMC8177411; doi:10.1371/journal.pone.0251973)
Supplement: S6 Table — (PDF) [file pone.0251973.s006.pdf]

S6 Table. Effect of sperm incubation in capacitation medium with thapsigargin, gingerol or BAPTA-K+ on the AR (%)

Experimental groups	Experiment time	Incubation period				
		0 min	20 mins	30 mins	60 mins	180 mins
Capaciation media	First time	0	0.5	0.5	1	8.5
	Second time	0	0	1	0	8
	Third time	0	0	1	1	8
Thanpsigargin	First time	0	7.5	18	24	31.5
	Second time	0	0.5	15	14.5	21.5
	Third time	0	0.5	16	20	25
Gingerol	First time	0.5	0.5	0	2	11.5
	Second time	0	0	0	2.5	13
	Third time	0.5	0.5	0	2	12
BAPTA-K+	First time	0	0.5	0.5	0	0.5
	Second time	0	0.5	0.5	0.5	1.5
	Third time	0	0.5	0.5	0	1
