# Supplementary material for: ACRBP (Sp32) is involved in priming sperm for the acrosome reaction and the binding of sperm to the zona pellucida in a porcine model
Source: PLoS One. 2021 Jun 4;16(6):e0251973. doi: 10.1371/journal.pone.0251973 (PMC8177411; doi:10.1371/journal.pone.0251973)
Supplement: Attachment — Submitted filename: renamed_5e534.docx [file pone.0251973.s007.docx]

Re: PLOS ONE Decision: Revision required [PONE-D-20-10428] [EMID:df198b69de0b9df3]

Title: ACRBP (Sp32) is involved in priming sperm for the acrosome reaction and the binding of sperm to the zona pellucida in a porcine model

Authors: Yoku Kato, Satheesh Kumar, Christian Lessard, Janice L Bailey

Dear Professor Drevet,

Thank you for sending your reviews. We appreciate the helpful suggestions offered by the editor and reviewer as their comments were valuable for improving this manuscript. We have modified our manuscript for PLOS ONE in accordance with comments from editor and reviewers and hereby resubmit the revised manuscript for consideration. In addition, one supporting information file of the additional data was added. These changes have addressed all of critiques of the reviewers. All revisions are listed as followed.

As the reviewer mentioned the grammatical errors, the attached paper has been carefully reviewed by an experienced editor whose first language is English and who is authorized as an official marker of International English Language Testing System and specialized in the editing of papers written by scientists whose native language is not English.

We hope that the revised version of our manuscript is now suitable for publication in PLOS ONE and we look forward to hearing from you at your earliest convenience.

Yours sincerely,

Yoku Kato, Ph.D

Centre de recherche en reproduction,

développement et santé intergénérationnelle,

Département des sciences animales,

Université Laval, Ste-Foy, Québec, Canada

Tel: +81-90-4169-3285

E-mail; hiroisora2001@yahoo.co.jp

Janice L. Bailey, Ph.D

Fonds de recherche du Québec

140, Grande Allée Est, bureau 450

Québec (Québec) G1R 5M8

Tel: +1-418 643-3230

E-mail: janice.bailey@frq.gouv.qc.ca

[Comments and Questions]

1. Reviewer #2, MAJOR COMMENTS: One first point in my major comments was that the fact that antibodies linked on the sperm surface inhibit sperm zona-pellucida binding is not a guarantee that the targeted protein is a key player in the fertilization process, as was proven in the past for different candidate (such as the Crisps proteins). This point has not been discussed by the authors. Are there studies with ACRBP gene invalidation corroborating the putative ZP-binding function of ACRBP?

Reply: Revised Introduction section. Please note L108-112:

“In vitro fertilization (IVF) assays using cumulus-intact oocytes indicated a clear reduction in the capacity of sperm produced by mice lacking the ACRBP gene to fertilize the oocytes. The fertilization rate in the deficient sperm was less than 10% of that of normal sperm. The abilities of the deficient sperm to bind to the zona pellucida and to fuse with the oolemma were significantly decreased [34].”

2. Reviewer #2, MAJOR COMMENTS: In the first review, the reviewer mentioned “the fact that antibodies linked on the sperm surface inhibit sperm zona-pellucida binding is not a guarantee that the targeted protein is a key player in the fertilization process, as was proven in past for different candidate (such as the Crisp proteins). The most interesting action o ACRBP shown in this paper may thus be the potential interaction with SERCA. In this regard, even though the authors mention in their conclusion that “Future experiments are necessary to assess how ACRBP promotes the activation of the Ca2+ channels on the sperm surface”, it would strengthen the current work to show the co-localisation of these two proteins during the capacitation process. Maybe the authors could perform simple co-localization IF studies using confocal microscopy or proximity ligation assays? This would not impeach a more mechanistic study as proposed in the conclusion (with co-IP and intracellular calcium measurements maybe?). “

In the second review, the same reviewer’s comments was that “Maybe the authors could perform simple co-localization IF studies using confocal microscopy or proximity ligation assays?” in order to show the co-localisation of the two studied proteins during the capacitation process. The authors performed IF staining of each protein in two independent experiments. The results only show that each protein is present in the acrosome part. Co-localisation studies usually involve co-staining if you use IF, and confocal microscopy with image analysis to prove that the two signals are indeed co-localized. Proximity ligation assay is another possible option, maybe more difficult to perform. In my opinion the authors did not answer that point in a satisfactory way. If the authors believe that the co-localisation is not a crucial point to sustain the function of these proteins, they need to argue in that sense. Furthermore, the investigation of the co-localisation has not been considered during the capacitation process, as was mentioned in my first report”.

Reply: Thank you very much for these suggestions. Although our ability to conduct the exact experiments suggested by Reviewer #2 is impeded by the pandemic, we have I added another experiment to show the interaction between ACRBP and SERCA. Please note L696-L729 and Figures 3 and 4.

3. Reviewer #2, MAJOR COMMENTS: In their answer concerning the discrepancies in the data relating the percentages of acrosome reacted sperm (see Tables 4 and 5 of the revised manuscript), the authors mention that the season is the cause. Nothing has been added in the text. Maybe this point is an evidence for researchers specialized in pig semen, but this is not the case for others although they may work in the reproductive field.

   Reply: I added the sentence in the discussion section. Please note L510-515 in the Results section.
4. Reviewer #2, MAJOR COMMENTS: I suggested two references concerning the identity of identified tyrosine-phosphorylated proteins during capacitation. The authors used other ones, which is not the problem. My concern is that they still mention “only a few phosphorylated proteins, which are hexokinase in the sperm head and the a-kinase-anchoring protein in the flagellum, have been formally identified [43]”. If they mean in porcine, it needs to be clearly stated, because in the two references I have proposed, other proteins were identified (45 proteins in Chung et al., 2014 using phopsphoproteomics analysis). Maybe the authors consider that these proteins were not “formally identified” but they have to argue that point.

   Reply: Thank you. The two suggested references are included. Please note L606-609 in the Discussion.
5. Reviewer #2, MAJOR COMMENTS: There are very numerous spelling or grammar errors in the added portions of the text, and even a reference in a place where it should not be (lines 324-327). The authors should be more careful when submitting a paper for review. As an indication, I noticed errors in lines 282, 284, 293, 295, 304,307,309, 320, 321, 324-327, 332.334, 361, 363, 400, legend of table 3, 440, 445 (discrepancy between the text and the table legend), 520, 536-537, 563, 564, 573, 575, 668, and probably others that I missed.

   Reply: As the reviewer mentioned the spelling and grammatical errors, the attached paper has been carefully reviewed by an experienced editor whose first language is English and who is authorized as an official marker of International English Language Testing System and specialized in the editing of papers written by scientists whose native language is not English. Before we submitted the last revision, the editor had corrected the grammatical errors and spelling thoroughly. Please note the lines 27, 28, 29, 32, 34, 37, 69, 74, 75, 77, 79, 80, 84, 88, 98, 99, 101, 102, 104, 114, 115, 120, 121, 151, 152, 153, 156, 157, 166, 172, 174, 175, 179, 183, 184, 185, 186, 193, 198, 199, 200, 201, 202, 206, 207, 213, 217, 218, 227,228, 235, 241, 253, 257, 258, 264, 267, 274, 276, 279, 281, 283, 284, 285, 286, 293, 295, 296, 297, 298, 299, 301, 306, 314, 315, 326, 333, 337, 342, 343, 344, 345, 347, 352, 356, 358, 360, 363, 364, 366, 367, 369, 370, 375, 376, 385, 388, 391, 392, 393, 424, 425, 426, 427, 428, 429, 430, 431, 434, 435, 458, 467(discrepancy between the text and the table legend), 468, 469, 471, 471, 472, 473, 504, 505, 507, 542, 543, 549, 550, 556, 559, 564, 565, 581, 582, 584, 585, 586, 592, 594, 596, 598, 599, 600, 602, 606, 611, 614, 619, 620, 623, 624, 625, 626, 627, 636, 637, 638, 639, 640, 641, 642, 643, 644, 646, 648, 649, 650, 651, 652, 653, 654, 655, 656, 657, 658, 659, 660, 661, 662, 663, 664, 665, 666, 667, 668, 669, 670, 671, 672, 673, 674, 675, 678, 679, 680, 681, 682, 683, 684, 685, 686, 688, 689, 690, 692, 693, 694, 694, 695, 731, 733, 746, 748, Table 1, Table 3, and Table 4. In addition, I realized that the reference on the line 324 and 327 in the previous version of paper is incorrect. The place was corrected and please see the line 326.
